# Confocal microscopy 3D imaging of diesel particulate matter

**DOI:** 10.1007/s11356-021-14025-y

**Published:** 2021-04-23

**Authors:** Lisa Miyashita, Gary Foley, Ian Gill, Gavin Gillmore, Jonathan Grigg, David Wertheim

**Affiliations:** 1 Centre for Genomics and Child Health, Blizard Institute, Queen Mary University of London, London, UK; 2 Faculty of Science, Engineering and Computing, Kingston University, Surrey, KT1 2EE UK; 3 Present Address: School of Science, Bath Spa University, Bath, UK

**Keywords:** Particulate matter, Diesel particulate matter, Confocal microscopy, 3D microscope imaging

## Abstract

To date, diesel particulate matter (DPM) has been described as aggregates of spherule particles with a smooth appearing surface. We have used a new colour confocal microscope imaging method to study the 3D shape of diesel particulate matter (DPM); we observed that the particles can have sharp jagged appearing edges and consistent with these findings, 2D light microscopy demonstrated that DPM adheres to human lung epithelial cells. Importantly, the slide preparation and confocal microscopy method applied avoids possible alteration to the particles’ surfaces and enables colour 3D visualisation of the particles. From twenty-one PM_10_ particles, the mean (standard deviation) major axis length was 5.6 (2.25) μm with corresponding values for the minor axis length of 3.8 (1.25) μm. These new findings may help explain why air pollution particulate matter (PM) has the ability to infiltrate human airway cells, potentially leading to respiratory tract, cardiovascular and neurological disease.

## Introduction

It has been suggested that air pollution is a major cause of premature death and is a recognised risk factor leading to human respiratory disease (Anderson et al. 2012, World Health Organization 2018, Khomenko et al. 2021). A major component of air pollution is in the form of particulate matter (PM). The impact of air pollution PM on human health is not restricted to effects in the lungs, with several studies identifying a link with heart disease, neurological disease and adverse pregnancy outcomes (Anderson et al. 2012, Klepac et al. 2018, Ren et al. 2019, Wu et al. 2019). Air pollution–derived PM has a number of natural causes such as wildfires and volcanic eruptions but in urban areas, anthropogenic sources such as emissions from traffic predominate (Grigg 2012). In particular, emissions from diesel engines are considered to be one of the largest contributors to environmental pollution (Lloyd and Cackette 2001). Overall, PM released from combustible sources is primarily composed of black carbon (Anderson et al. 2012) and is grouped into three main categories based on aerodynamic diameter: PM_10_ (< 10 μm), PM_2.5_ (< 2.5 μm) and ultrafine PM (< 1 μm). The size determines its aerodynamic characteristics and consequently its capacity to penetrate the alveolar wall and enter the bloodstream (Brugha et al. 2014). Evidence of combustion-derived PM has recently been reported in both brain and heart tissue (Maher et al. 2016, Calderon-Garciduenas et al. 2019).

Numerous studies have focused on the immunological effect of air pollution–derived PM; however, research defining the morphology of this PM is lacking. It was demonstrated that non-pollution-derived particles with a more defined, sharper edge displaced lung surfactant to a larger degree than those with a spherical composition, allowing these particles to be more readily internalised by the lung epithelium (Gerber et al. 2006). To date, combustion-derived PM is reported to consist of aggregates of carbonaceous spherules with a smooth appearing surface (Yang et al. 2016, Zeb et al. 2018). Recently, a novel method was developed to visually assess volcanic PM in 3D by high-resolution laser scanning confocal microscopy (Wertheim et al. 2017). In contrast to the previously suggested spherical nature of diesel PM, the morphology of some volcanic particulates was shown to be jagged.

To help understand the morphology of diesel particulate matter (DPM) also known as diesel exhaust particulate (DEP) and mode of action on lung epithelial cell invasion, our study aimed to investigate the 3D structure of DPM and its interaction with human lung epithelial cells.

## Materials and methods

DPM was acquired in powder form from the National Institute of Standards and Technology (NIST,SRM2975,USA). Double-sided adhesive carbon disks (12mm, Agar Scientific Ltd., UK) were adhered to microscope slides (VWR International, UK). DPM was sieved through a mesh filter (50μm, VWR) onto the carbon disk slides to prevent aggregation of particles. DPM slides were then imaged using a LEXT OLS4100 confocal microscope (Olympus Corporation, Japan) with a 405-nm laser. Images with resolution 1024 × 1024 pixels were taken using a ×100 objective lens (numerical aperture 0.95) and collected using the fine mode setting. Imaging threshold parameters were set by adjusting the upper limit to just above the top of the particles and the lower limit to just below the level of the adhesive disk (Wertheim et al. 2017). Particle size was measured using the Olympus OLS4100 microscope system software (Olympus Corporation, Japan). For size measurements, particles were considered as approximately elliptical and the longest 2D axis was termed ‘major axis’ and the perpendicular axis termed ‘minor axis’; the maximum 3D height was determined using a profile tool in the software. Descriptive statistics of particle size data were calculated using Minitab v19 (Minitab Inc., USA).

A549 adenocarcinomic human alveolar epithelial cells were seeded into Nunc® chamber well slides (Merck, UK) overnight and incubated with 10μg/ml DPM for 2 h, thoroughly washed and stained (Hemacolor®, VWR international). Fifty images with resolution 746 × 500 pixels were taken at random by light microscopy (Nikon Eclipse 80i) using a ×100 objective lens.

## Results

**Fig. 1:**
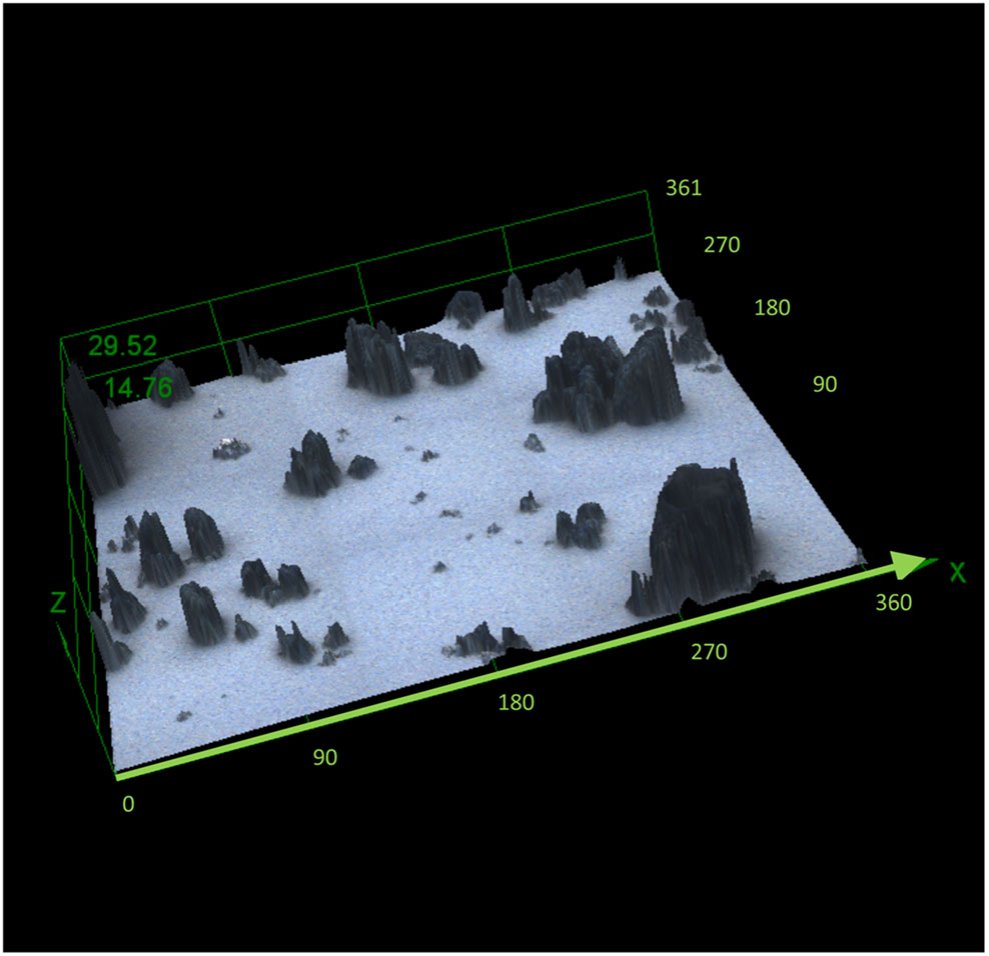
View of 9 images stitched together to form a region with dimensions 360 × 360 × 30 μm; each of the 9 images was acquired using a ×100 objective lens. For clarity, the image has a z axis factor of 2 which magnifies the relative z axis component visualisation

**Fig. 2:**
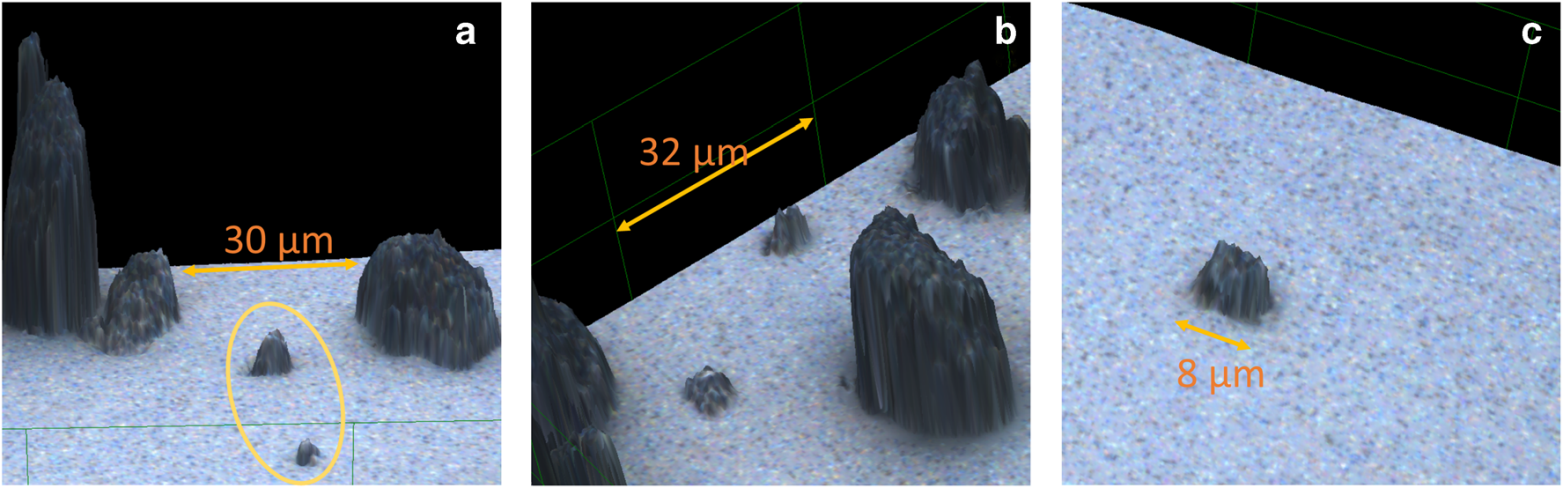
**a** A close-up of two PM_10_ particles in orange oval (4.8 by 4.4, max height 2.8 and 2.8 by 2.4, max height 1.3 μm) surrounded by three large particle aggregates with max heights of 19.9, 4.4 and 7.6 μm (left to right); **b** Close-up of one of the constituent stitched images in Fig. 1 with two PM_10_ particles with major and minor axis dimensions of 5.6 by 4.5 (max height 3.1) and 5.8 by 4.9 (max height 2.2) μm; one of the particles in particular appears to have a sharp protruding surface; the orange scale bar indicates linear distance of 32 μm; **c** Image obtained with a ×100 objective lens is a zoomed in close-up showing another sharp appearing particle with major and minor axis dimensions of 7.7 by 7.0 (max height 3.7) μm. For clarity, all images have a *z* axis factor of 2 which magnifies the relative *z* axis component visualisation

Images were successfully acquired with the confocal microscope in 2D and 3D from the slides and demonstrated a variety of DPM shapes and sizes. A stitched image consisting of 9 adjacent partially overlapping images in Fig. 1 demonstrates the heterogeneous nature of particle morphology over a larger area. The ≤PM_10_ particles frequently had sharp, jagged appearing edges; the images show comparison with larger particulate aggregates (Fig. 2a and 2b) and an individual particle (Fig. 2c). Some particles imaged may in part consist of agglomeration of fine particles. The images revealed particles of a comparable nature in colour and shape, making the likelihood of contamination low. As the microscope illumination and imaging are from above the sample and the DPM is opaque, the shape of the bottom surface on the adhesive carbon disk could not be discerned in detail.

**Fig. 3:**
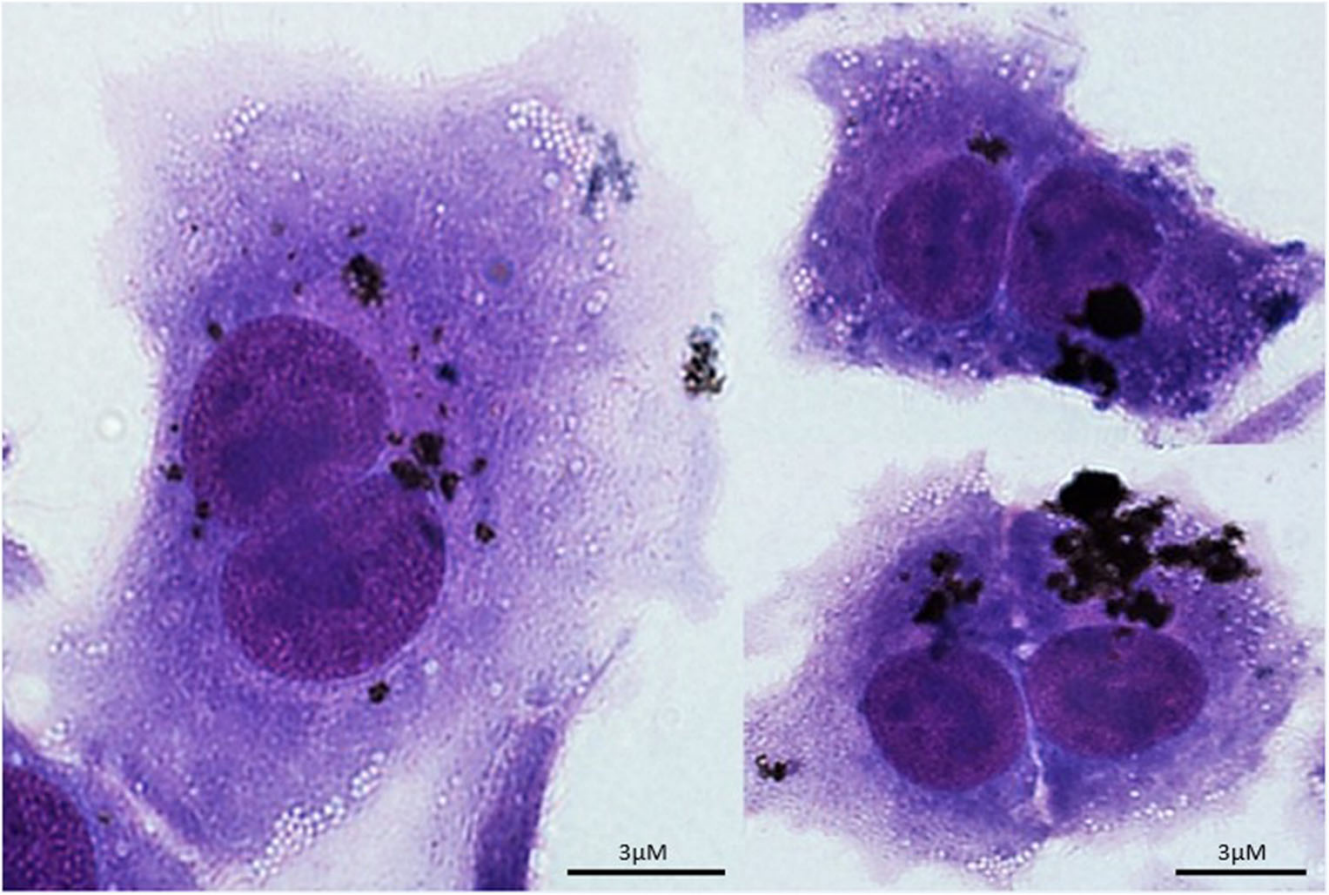
Image of six A549 lung epithelial cells exposed to 10 μg/ml DPM. DPM is observed to be adherent to cells

From measurements of twenty-one ≤PM_10_ particles, the mean (standard deviation) major axis length was 5.6 (2.25)μm with corresponding values for the minor axis length 3.8 (1.25)μm and the ratio major/minor was 1.5 (0.46); the ratio also suggests particles 2D cross-section are often not circular. Culture of A549 lung epithelial cells with 10μg/ml DPM in vitro, followed by vigorous washing to remove unbound particles, demonstrated that DPM can exhibit adherence to the cells (Fig. 3).

## Discussion

Epidemiological studies have demonstrated a likely link between PM concentration exposure and the onset of respiratory, cardiovascular and neurological disease (Anderson et al. 2012). For example, exposure to elevated PM concentration is suggested to be related to the incidence of hospital admission (Wei et al. 2019, de Aguiar Pontes Pamplona et al. 2020) and adverse health effects in conditions such as respiratory disease for instance asthma (Grigg 2012, Thurston et al. 2020), heart disease (Tian et al. 2019, de Aguiar Pontes Pamplona et al. 2020, Chen et al. 2020) and stroke (Huang et al. 2019); recently, it has also been suggested that exposure to high levels of particulate matter may be associated with increased blood pressure in adults (Xu et al. 2020). An increased incidence of health-related disease, likely associated with high levels of ambient PM, can also occur following volcanic eruptions (Forbes et al. 2003, Oudin et al. 2013, Carlsen et al. 2015) and forest fires (Dennekamp and Abramson 2011).

The underlying mechanisms that drive elevated levels of ambient PM to increase disease onset or exacerbation remain unclear. If this were better understood, it could help in the development of novel strategies to ameliorate the adverse effects of elevated PM that result from human activity and natural events. For example, studies have demonstrated the ability of PM to be internalised by, or tightly adhere to airway epithelial cells (Colasanti et al. 2018), as well as lung tissue (Mäkelä et al. 2019); however, the mode of action is yet to be fully elucidated. Furthermore, PM from various sources are likely to invoke differences in disease severity. This is demonstrated in a study that identified Baltimore PM to induce a much greater inflammatory response compared to that in New York City (Gour et al. 2018). Defining the inflammatory profile of PM constituents may therefore be an important factor in predicting adverse health effects.

Several methods of imaging aerosol particles, other than confocal microscopy, have been listed by Li et al. (2016). Indeed previous studies of the appearance of particulate matter have generally used 2D imaging such as investigations based on optical microscopy (Davis and Jixiang 2000, Tian et al. 2017, Koval et al. 2018), scanning electron microscopy (SEM) (Yang et al. 2016, Selley et al. 2020), transmission electron microscopy (TEM) (Bérubé et al. 1999, Chandler et al. 2007, Liati et al. 2012) or for 3D imaging of particles, stereo SEM (Mills and Rose 2010). Previous studies of diesel exhaust particulate morphology have often used SEM or TEM (Figler et al. 1996, Liati et al. 2012, Liati et al. 2013, Baldelli et al. 2020). Stereo SEM can generate 3D reconstruction of particles with such techniques being reliant on homologous points and interpolation (Proussevitch et al. 2011); furthermore, as electrons are used to image the sample in SEM and TEM, there is no simultaneous overlaid true colour image. However, our laser scanning confocal microscopy technique allows direct 3D measurement of particles together with true colour visualisation.

In contrast to a previous study that depicted haze-related urban PM as spherical based on 2D SEM (Zeb et al. 2018), we have shown using 3D confocal imaging that DPM can contain particles with sharp appearing edges. Diesel emission particles can vary in size from fine particles less than 100nm to several micrometers (Liati et al. 2013, Rocha and Corrêa 2018); in view of the range of DPM size, it is possible that at least some particles imaged with confocal microscopy may consist of fine particle agglomeration. A recent study has reported that brake dust particles can also have jagged edges (Selley et al. 2020). DPM matter is created by controlled spontaneous combustion at high temperatures within the engine chamber, leading to the formation of fragments with varying morphology, some of which have sharp appearing edges. These jagged edges may help to explain the observed adherence of DPM to lung epithelial cells (Fig. 3) in our study.

More research is needed to identify the direct effect morphology has on cell integrity, and the observed adherence of DPM to alveolar epithelial cells cannot be explained by morphology alone. However, as sharp non-pollution-derived particles are more readily internalised by lung epithelium compared to those more spherical (Gerber et al. 2006), it is plausible that the observed jagged morphology of DPM has similar effects in the airways. Furthermore, sharp-edged particles are reported to significantly enhance cytokine release compared to smooth, spherule particles of the same size, suggesting that morphology has an important role in the inflammatory potential of PM (Lebre et al. 2017). In addition, it has been suggested that adverse health effects are associated with the interaction of PM_2.5_ surface groups with biomolecules in lung fluid (Zhou et al. 2016).

We postulate that these sharp edges more readily present the airway immune system with foreign epitopes, initiating cellular uptake by alveolar macrophages (AM) which have been shown to phagocytose air pollution–derived PM in a similar manner to bacteria (Brugha et al. 2014). Furthermore, jagged edges combined with a small surface area may impact on cell integrity at the molecular level by either anchoring to the cell membrane and/or initiating van der Waals forces, both of which could induce cellular internalisation. These forces have previously been shown to have important biological functions and may represent a novel mechanism for cellular infiltration (Autumn et al. 2000).

Importantly, the methodology adopted in this study allows collection of colour 3D images of particles without the need for coating or mounting in a substrate thus avoiding possible alteration or damage to the surface of the particles. Fine particles, which are particularly detrimental to human health, previously observed with 2D microscopy can now be clearly visualised and defined in colour and in 3D.

## Conclusion

This study has used a novel method to show that microscopic DPM matter, which have been previously characterised as of spherical shape, includes particles with sharp appearing edges; this jagged appearance may aid the ability of particles to tightly adhere to epithelial cells and increase airway inflammation. We suggest that the observed particle morphology may enhance the capacity for air pollution–derived PM to enter the human body and subsequently cause adverse health effects. This straightforward novel methodology provides a means to compare air pollution–derived particulate morphology from various sources, to help define their inflammatory potential, to determine the consequent effect on cell integrity and, importantly, to contribute to the development of ameliorating measures.
